# Overweight, obesity and excessive weight gain in pregnancy as risk factors for adverse pregnancy outcomes: A narrative review

**DOI:** 10.1111/jhn.12999

**Published:** 2022-03-20

**Authors:** Simon C. Langley‐Evans, Jo Pearce, Sarah Ellis

**Affiliations:** ^1^ School of Biosciences University of Nottingham, Sutton Bonington Loughborough UK; ^2^ Food & Nutrition Subject Group Sheffield Hallam University Sheffield UK

**Keywords:** gestational diabetes, obesity, pre‐eclampsia, pregnancy, stillbirth

## Abstract

The global prevalence of overweight and obesity in pregnancy is rising and this represents a significant challenge for the management of pregnancy and delivery. Women who have a pre‐pregnancy body mass index greater than 25 kg m^–2^ are more likely than those with a body mass index in the ideal range (20–24.99 kg m^–2^) to have problems conceiving a child and are at greater risk of miscarriage and stillbirth. All pregnancy complications are more likely with overweight, obesity and excessive gestational weight gain, including those that pose a significant threat to the lives of mothers and babies. Labour complications arise more often when pregnancies are complicated by overweight and obesity. Pregnancy is a stage of life when women have greater openness to messages about their lifestyle and health. It is also a time when they come into greater contact with health professionals. Currently management of pregnancy weight gain and the impact of overweight tends to be poor, although a number of research studies have demonstrated that appropriate interventions based around dietary change can be effective in controlling weight gain and reducing the risk of pregnancy complications. The development of individualised and flexible plans for avoiding adverse outcomes of obesity in pregnancy will require investment in training of health professionals and better integration into normal antenatal care.

## Highlights

- Overweight and obesity before pregnancy and excessive gestational weight gain are major determinants of risk for pregnancy loss, gestational diabetes, hypertensive conditions, labour complications and maternal death.
- Pregnancy is regarded as a teachable moment when women are at their most receptive to messages about their health. However, unclear guidance on diet and physical activity, weight stigma from health professionals, inexperience and reluctance among professionals about raising issues about weight, and stretched resources put the health of women and babies at risk.
- Excessive weight gain in pregnancy and post‐partum weight retention compromise future fertility and increase risk for future pregnancies.
- Large randomised controlled trials have had little success in addressing excessive gestational weight gain or antenatal complications. Individualised, culturally sensitive and responsive interventions appear to have greater success.

## Graphical abstract

Overweight and obesity complicate up to two‐thirds of pregnancies, and increase risk of gestational diabetes, hypertension, pre‐eclampsia, and maternal and fetal death. Interventions to reduce risk can be effective but will require improved protocols to discuss weight with pregnant women and enhanced training for midwives to deliver appropriate guidance on lifestyle change.

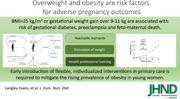

## INTRODUCTION

Modern medical care has made pregnancy and childbirth relatively safe for women who live in developed countries. Across the Global North the maternal death rate is less than 1 per 10,000 births and rates of stillbirth and late fetal death rates are between four and six per 1000 births.^1^ Improvements in outcomes since the 1960s have been driven by a range of factors, including high standards of hygiene and sanitation and improved nutrition, but, most importantly, women's control over reproduction.^2^ Because approximately 60% of pregnancies are now planned in advance, there is an opportunity for women and their partners to make lifestyle changes that promote better health in pregnancy and reduce risk of poor pregnancy outcomes.^3^

The relative safety of pregnancy and childbirth for women in high‐income countries is a major benefit of the medicalisation of pregnancy, characterised by antenatal surveillance and intervention. Of course, this is not true of countries in the Global South where pregnancy and childbirth‐related complications remain the major cause of death for young women. Where women benefit from advances in obstetric care, there are concerns that the medical management of pregnancy has become too intrusive and that the benefits of close surveillance and early intervention, particularly in labour, do not justify the associated cost and the loss of autonomy for women.^4^, ^5^, ^6^ Against this background, it is surprising that dietary change and weight management is not a fixed feature of pregnancy care. Dietary advice is loose and in addition to being given a list of things to avoid (potential sources of food pathogens, liver, oily fish, alcohol and caffeine) women are merely advised to consume a healthy ‘balanced’ diet. Given the low quality of the western diet and the current prevalence of overweight and obesity, this approach is unlikely to have any efficacy. For many women, there is no advice given on weight gain until they are already pregnant and even then, it is lacking in quality. As this review will describe, the avoidance of overweight and obesity should be the highest priority for women who are considering becoming pregnant because excessive body fat is the single greatest risk factor for poor pregnancy outcomes and pregnancy complications.

The prevalence of overweight and obesity is highly variable across the world, with the highest rates in women observed in the Pacific island nations, the Caribbean and the Middle East. It is estimated that, globally, there are close to 39 million pregnancies per year complicated by maternal obesity^7^ and, in some countries, the estimated prevalence of overweight and obesity in pregnancy is over 60% (South Africa 64%, Mexico 65%, USA 55%–63%).^7^, ^8^ In England, the combined prevalence of overweight and obesity is 35% among 16–24‐year‐old women, rising to 61% among 35–44 year‐olds, highlighting the high level of potential risk among women of reproductive age.^9^ The highest rates of antenatal obesity are observed in areas of high deprivation, among older mothers and in minority ethnic groups.^10^ Women in the UK who are Black (odds ration [OR] = 1.70, 95% confidence interval [CI] = 1.62–1.78) or South Asian (combined OR = 1.72, 95% CI = 0.66–1.79) are reported to be more likely to be living with obesity than white women.^11^ It is of course well recognised that rates of obesity have been rising quickly over the last two to three decades and, increasingly, pregnancy is being complicated by extreme or morbid obesity. In the UK, it has been estimated that approximately 1 in 1000 births are to women with a body mass index (BMI) > 50 kg m^–2^, whereas, in Australia, a super‐obesity prevalence of 2.1 per 1000 births was noted.^12^, ^13^

**Figure 1:**
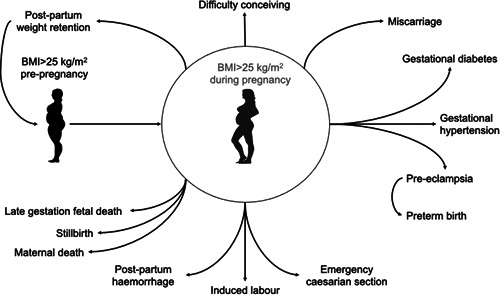
Obesity in pregnancy is a risk factor for adverse outcomes. BMI, body mass index. Adapted from Langley‐Evans^16^

This review discusses the implications of overweight and obesity for pregnancy complications and outcomes. As shown in Figure 1, overweight is a significant risk factor for infertility, loss of pregnancy, pregnancy complications, complications in labour, and fetal and maternal death. All of these risks are also associated with excessive weight gain during pregnancy. Because women can often struggle to lose weight gained in pregnancy,^14^ excessive pregnancy weight gain can also put future pregnancies at risk of poor outcomes.^15^ Greater interpregnancy weight gain is also a factor in establishing greater risk for future pregnancies.^16^

Gestational weight gain (GWG) comprises both fetal and maternal components. On the fetal side, there is mass laid down to develop the placenta, amniotic fluid and fetal tissues. Women also gain weight because of an increase in body water and blood volume to support perfusion of the placenta, deposition of tissue in the breasts in readiness for feeding post‐partum, expansion of the uterus and deposition of fat in stores.^17^ Achieving a satisfactory GWG is extremely important for successful completion of gestation. In some parts of the world, pregnant women are given some guidance on what would be appropriate GWG, whereas, in the UK, such advice is not available. In terms of evaluating GWG for research purposes, it is generally accepted that the United States Institute of Medicine guidelines are appropriate (Table 1).^18^ Appropriate ranges of weight gain are dependent upon women's BMI going into pregnancy.^18^ Women who are underweight prior to pregnancy need to gain more weight to avoid complications associated with inadequate GWG, whereas obese women should control weight gain to avoid excessive GWG. Greater weight gain is expected for women who are carrying twins, reflecting the greater amount of fetal and placental tissue to be laid down (Table 1).^19^

**Table 1:** Recommendations for weight gain in pregnancy are related to pre‐pregnancy body mass index

Body mass index at conception (kg m^–2^)	Optimal weight gain (kg) for singleton pregnancy	Optimal weight gain (kg) for twin pregnancy
Underweight < 18.5	13–18	23–28
Normal weight 18.5–24.9	11–16	18–25
Overweight 25–29.9	7–11	17–21
Obese > 30	5–9	13–17

Data Sources: Luke^19^; Institute of Medicine^18^. Optimal weight gain ranges are those associated with favourable pregnancy outcomes for mother and fetus and which lead to a birth weight between 3.1 and 3.6 kg.

**Figure 2:**
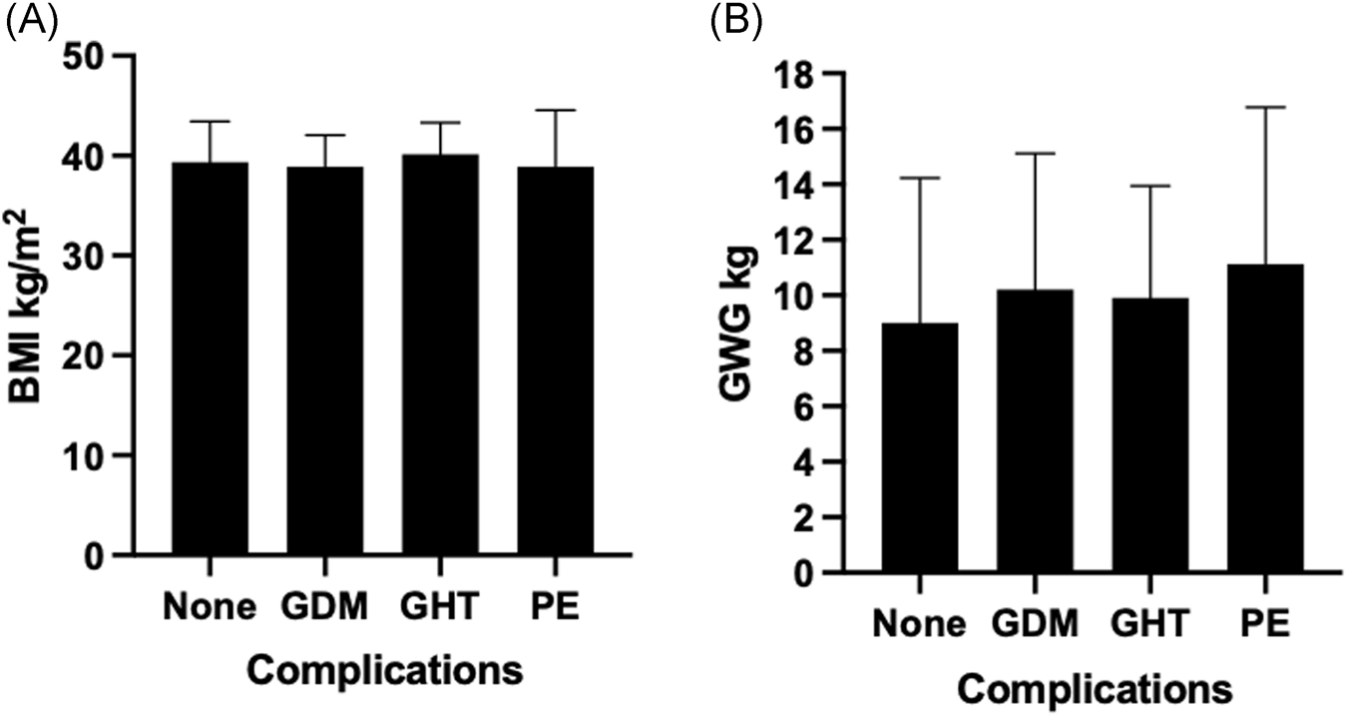
Early pregnancy body mass index (BMI) and gestational weight gain in relation to pregnancy complications (a) Distribution of BMI among severely obese pregnant women. (b) Distribution of gestational weight gain among severely obese pregnant women. All women were of BMI ≥ 35 kg m^–2^ at antenatal booking (*n* = 387). Gestational weight gain (GWG) was determined as weight gain between booking and 36 weeks of gestation. Data are shown as median and interquartile ranges. No complications *n* = 291 (75% of cohort); gestational diabetes (GDM), *n* = 45 (11.5%); gestational hypertension (GHT), *n* = 37 (9.5%); pre‐eclampsia (PE), *n* = 16 (4%)

Although overweight and obesity greatly increase the risk of adverse outcomes in pregnancy, it is important to appreciate that the majority of women with BMI > 25 kg m^–2^ will have normal, uncomplicated pregnancies. Among 387 British women with a BMI in excess of 35 kg m^–2^ at booking, 75% went through a full gestation without developing any of the major complications of pregnancy (Figure 2). Similarly Relph et al.^20^ found that among more than 115,000 Canadian women with BMI > 30 kg m^–2^, with no underlying morbidities, nearly 60% had a normal pregnancy. The major complications of pregnancy are relatively uncommon events. One in 23 women in the UK develop gestational diabetes (GDM); one in 18 develop pre‐eclampsia (PE) (of which only a one‐third will have severe PE) and one in 13 give birth preterm (before 37 weeks; of which 10% do so as a result of PE). Although there are significant numbers of women affected each year and there are major concerns about health at the population level, the risk faced by individual women living with obesity remains small.^21^

## OBESITY AND INFERTILITY

Body fatness is the main lifestyle‐related factor that has effects on reproductive health in women. Both underweight and obesity are associated with menstrual cycle disorders including anovulation, amenorrhea and oliogorrhea. This relationship is mediated through leptin, which is produced by adipose tissue. Leptin concentrations are directly proportional to the amount of adipose tissue in the body and this hormone has a permissive effect on the secretion of gonadotrophin‐releasing hormone from the hypothalamus and luteinising hormone and follicle‐stimulating hormone from the pituitary. In obesity, women develop leptin resistance leading to cycle disorders.^22^ Rich‐Edwards et al.^23^ found that there was a U‐shaped relationship between BMI and ovulatory disorders, with 25% of such disorders in the US population being explained by obesity. Among women with no obvious menstrual cycle disorders, overweight and obesity delay conception in women who are trying to become pregnant.^24^ In women who have bariatric surgery to treat obesity, fertility improves and pregnancy outcomes are favourable.^25^ In women who are undergoing assisted reproductive therapies (ART), obesity reduces the efficacy of those treatments. Comparing women with BMI > 30 kg m^–2^ with those at 18.5–24.9 kg m^–2^, the odds of a live birth were significantly reduced (relative risk [RR] = 0.85, 95% CI = 0.82–0.87).^26^ Weight loss is generally advised for obese women prior to commencing ART.

Alongside infertility that is driven by leptin resistance, obesity is the major risk factor for polycystic ovary syndrome (PCOS). PCOS is one of the more common fertility issues in women and is associated with anovulation and irregular menstrual cycles. The cause of fertility problems in PCOS is elevated androgen concentrations, although this is secondary to insulin resistance.^27^ Although more common in women living with obesity,^28^, ^29^ PCOS also occurs in women who are not obese and risk is related to visceral fat mass.^30^, ^31^ PCOS and its associated menstrual cycle disorders are readily treated with metformin to improve insulin sensitivity, or through weight loss of approximately 5% body weight.^32^ Although a number of studies have evaluated whether low carbohydrate diets or similarly restricted approaches have particular efficacy in restoring fertility in PCOS, standard weight loss strategies (exercise and energy restriction) appear to be the most effective and straightforward approach.^33^

## MISCARRIAGE, STILLBIRTH AND MATERNAL DEATH

Maternal BMI is a known determinant of the risk of spontaneous miscarriage in the first trimester of pregnancy. Both extremes of the BMI range are considered to increase risk, although the evidence of an adverse effect of underweight may be more robustly supported by the literature than overweight.^34^, ^35^ Both low concentrations of leptin and leptin resistance may play a role in miscarriage because this hormone has a role in embryonic implantation and the establishment of the placenta.^36^ Although Bracken and Langhe^37^ found no relationship between obesity and miscarriage, large studies of Asian populations indicate a modest but significant risk. Pan et al.^38^ investigated more than half a million pregnancies in China and found that, although overweight was not a risk factor for miscarriage, BMI > 28 kg m^–2^ (cut‐off for obesity in Asians) was associated with a 16% greater risk. Similar findings were reported by Haque et al.,^39^ who noted 8% greater risk with overweight and 26% greater risk with obesity. Among women undergoing ART there are greater rates of miscarriage with obesity.^40^, ^41^

The relationship between maternal BMI, GWG and risk of stillbirth is more complex than is often reported. Inadequate weight gain or weight loss in pregnancy have been demonstrated to increase stillbirth risk but, in women who were morbidly obese going into pregnancy, some weight loss in the second trimester reduced risk by 14%.^42^ Johansson et al.^43^ reported a greater risk of stillbirth with excessive GWG but only in women of normal weight pre‐pregnancy. Other studies suggest that stillbirth is more likely in pregnancies complicated by overweight^44^ or obesity.^39^, ^42^, ^45^ In South Asian women, obesity increased risk substantially (OR = 1.46, 95% CI = 1.27–1.67).^39^ Excessive GWG doubled stillbirth risk in women living with obesity in the study of Yao et al.^42^, ^45^ and the risk associated with obesity increased substantially in women whose pregnancies exceed 39 weeks in duration. A systematic review and meta‐analysis including over 16,000 stillbirths across 38 studies concluded that for every increase in BMI of 5 kg m^–2^ above the ideal range, the odds of stillbirth increased by 24% (OR = 1.24, 95% CI = 1.18–1.30).^46^

Severe obesity is a risk factor for maternal death in the perinatal period. An analysis of the outcomes of 571,000 pregnancies in New York City (2008–2012) found that death was significantly more likely in women with BMI  > 35 kg m^–2^ than in women of ideal weight.^47^ The level of risk increased with severity of obesity (BMI = 35–39.9 kg m^–2^, RR = 1.14, 95% CI = 1.05–1.23; BMI = 40–49.9 kg m^–2^, RR = 1.34, 95% CI = 1.21–1.49; BMI > 50 kg m^–2^, RR = 1.99, 95% CI = 1.57–2.54).^47^ Knight et al.^48^ considered all 209 UK women who died during pregnancy and up to 6 weeks post‐partum between 2015 and 2017. Cardiovascular complications were the biggest cause of maternal death and 55% of such deaths occurred in women who were overweight or obese. A similar evaluation of maternal mortality in France (2013–2015) concluded that overweight increased the risk of death by 60% and obesity more than tripled the risk, particularly for cardiovascular deaths.^49^ Maternal obesity was also a factor in the deaths of women who were infected with COVID‐19 during pregnancy, with more than double the risk of death in women with BMI > 30 kg m^–2^.^50^

## PREGNANCY COMPLICATIONS

Women who are overweight or obese are at generally higher risk of all complications of pregnancy. The major complications include GDM and PE, both of which present a significant risk of mortality for mother and baby. Less serious complications are experienced by a high proportion of pregnant women and include heartburn and symphysis pubis dysfunction (SPD). Although neither are a threat to successful delivery of a live baby, both are debilitating and chronic conditions in pregnancy. Obesity is a modifiable risk factor for SPD and possibly a pelvic girdle syndrome that persists beyond delivery of the baby.^51^ Denison et al.^52^ reported that, when comparing women with BMI > 30 kg m^–2^ with women with BMI under 25 kg m^–2^, the risk of SPD was almost four‐fold higher and risk of heartburn was increased by 2.65‐fold.

### Hypertensive disorders of pregnancy

Rising blood pressure is a normal feature of pregnancy and is generally not considered to be problematic. Blood pressure increases because renal function in pregnant women changes in order to handle a greater volume of blood and to perfuse the placenta. When the blood pressure increases to beyond the usual cut‐offs for hypertension (systolic 140 mmHg/diastolic 90 mmHg) in the last trimester of pregnancy, this is termed gestational hypertension (GHT), if there is no pre‐existing hypertension before conception and the condition arises no earlier than 20 weeks of gestation.^53^ In most cases, GHT is not a major problem but the condition needs to be closely monitored to detect progression to PE (regular proteinuria screening and additional antenatal appointments). If blood pressure increases to more than 160/110 mmHg, this is regarded as an obstetric emergency putting the lives of both mother and baby at risk.^54^, ^55^ Women with GHT are also at greater risk of all complications that may arise post‐partum, including haemorrhage.^56^ In addition to anti‐hypertensive medication, GHT is managed through lifestyle modification, including weight management and dietary sodium reduction.^55^ In a population of predominantly overweight and obese women, higher compliance with the DASH dietary pattern was associated with lower diastolic blood pressure and mean arterial blood pressure.^57^ GHT normally resolves within 3 months of giving birth but follow‐up monitoring is advised in case chronic hypertension develops.^53^, ^55^

Overweight and obesity are established risk factors for GHT.^52^, ^58^ In their very large cohort, Relph et al.^20^ observed that, although only 2.6% of normal weight women developed the condition, the prevalence was 4.7% in overweight women, 7.8% in obese and greater than 10% in severely obese women. A study that modelled the risk factors for GHT concluded that BMI > 25 kg m^–2^ was the biggest single predictor of developing GHT.^59^ Sormunen‐Harju et al.^60^ estimated that, compared to women with BMI 20 kg m^–2^, the risk of GHT was 2.3‐fold higher (95% CI 1.4–3.8) in women with BMI > 25 kg/m^2^. This risk rose markedly (42‐fold) in women who had previously had a pregnancy complicated by GHT. Excessive GWG is also a risk factor for GHT across all maternal BMI categories.^61^ GWG below guidelines was associated with a significant reduction in GHT risk in an analysis that drew on data from 18 cohort studies.^62^

**Figure 3:**
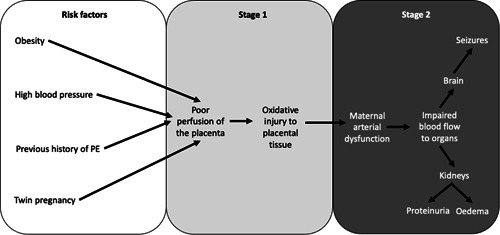
Factors that contribute to risk of pre‐eclampsia (PE) and disease progression

PE is an extremely dangerous condition that threatens the lives of both mother and fetus. It is characterised by the development of hypertension after 20 weeks of gestation and urinary protein excretion in excess of 300 mg/24 h.^63^ PE is caused by the development of arterial dysfunction in the placenta, which involves oxidative injury and an inflammatory response spreading beyond the placenta to impact upon all major organs in the mother.^64^ PE is a progressive condition that cannot be reversed or controlled and, without intervention, women are at risk of developing eclampsia. Eclampsia is the end stage of the PE disorder and is characterised by maternal seizures and coma as a result of oedema of the brain. Eclampsia can result in multiple organ failure, renal collapse, abruption of the placenta and death of both mother and baby (Figure 3). PE is the major cause of preterm delivery because the only viable treatment is to deliver the baby early by caesarean section.

There is a strong genetic component to PE and women who have had a pregnancy complicated by PE are likely to do so again in future pregnancies.^65^, ^66^, ^67^ Overweight and obesity are the next most important risk factors for PE and make the development of PE more likely because of insulin resistance (a promoter of vascular endothelial dysfunction) and a systemic pro‐inflammatory state as a result of the production of cytokines from adipose tissue. An analysis of a quarter of a million births in the Finnish Birth Registry showed that, among women under the age of 35 years, overweight increased risk of PE by 49%, whereas obesity increased risk by 2.45‐fold. In older women, the risk was significantly greater.^68^ A systematic review by He et al.^69^ found that overweight (OR = 1.71, 95% CI = 152–1.91) and obesity (OR = 2.48, 95% CI = 2.05–2.69) were major risk factors for PE. Among women who had bariatric surgery prior to pregnancy, risk of PE declined compared to obese women who did not have the surgery and compared to the same women in their pregnancies prior to surgery.^70^

### GDM

Pregnancy is an insulin resistant state in which changes to insulin signalling pathways suppress the uptake of glucose by the maternal muscle and liver.^71^ In the fed state, this serves to drive glucose into the fetal compartment as a substrate for growth. In the fasted state, it means that women mobilise more triglycerides, free fatty acids and ketones for fetal metabolism.^72^ Against this metabolic background, some women develop GDM, which has a number of potential adverse outcomes for babies when in utero and in the longer term. The most common outcome is macrosomia. Macrosomic infants weigh in excess of 4.5 kg at birth and this generally results in more caesarean sections because passage through the birth canal in a normal labour increases the risk of shoulder dystocia, bone fractures and subconjunctival haemorrhage.^73^ GDM increases the risk of congenital heart defects.^74^ and infants born to women with GDM are at greater risk of childhood obesity.^75^

The association between obesity and GDM is well documented. Systematic reviews and meta‐analyses of case–control and cohort studies indicate that obesity increases risk by more than threefold,^76^ and that excess visceral and central adiposity are greater risk factors than general obesity.^77^, ^78^ Both pre‐pregnancy and early pregnancy BMI is associated with risk of GDM, as is excessive GWG.^79^ Relph et al.^20^ found that, among over 700,000 Canadian women, risk of GDM increased with increasing BMI across the whole range. Although 2.8% of women with BMI under 18.5 kg m^–2^ developed the condition, 12.4% did so among women with BMI ≥ 50 kg m^–2^. A retrospective analysis of 4512 deliveries in Lagos found that excessive GWG was associated with greater risk of GDM (OR = 4.8, 95% CI = 1.93–12.62).^80^ Among food insecure Malaysian women, excessive GWG in the second trimester of pregnancy increased risk of GDM by almost 10‐fold.^81^

## LABOUR COMPLICATIONS

Obesity and excessive GWG increase risk of complications leading into, during and after labour, both directly and indirectly. In indirect terms, weight related conditions such as PE and GDM increase the likelihood of preterm delivery and delivery by caesarean section.^58^ In direct terms, labour is complicated by uterine dysfunction and caution on the part of medical professionals as they manage the labours of obese women.

Women who are overweight or obese are less likely than normal weight women to initiate and sustain spontaneous labour. Animal studies suggest that this is a consequence of reduced expression of uterine contractile proteins and production of labour‐inducing prostaglandins.^82^ As a result, these women are more likely to require labour induction.^83^ However, induction is less likely to succeed and obese women are more than three‐fold more likely than normal weight women to require an emergency caesarean after induction.^83^, ^84^, ^85^

Intervention in labour is overall more likely in obese women, who are less likely than women of ideal weight to have a spontaneous vaginal delivery.^86^ The most likely intervention is caesarean section (both elective and emergency).^58^, ^87^ and, to some extent, this is driven by medical staff seeking to minimise risk to the baby and mother. Women who are obese and whose labour is not progressing are less likely than women of ideal weight to be allowed to attempt a vaginal delivery with assistance from forceps or a vacuum cap (ventouse), but, when they are allowed to do so, appear to have better outcomes than ideal weight women.^88^, ^89^ With caesarean section recovery is slower and surgical complications are more likely with obesity.^90^, ^91^

Post‐partum haemorrhage occurs in up to 5% of women and is characterised by either heavy blood loss during delivery (in excess of 500 ml following vaginal delivery or 1 litre following a caesarean) or in the following days (as a result of placental retention, uterine atony or rupture). Obesity is one of the key risk factors for post‐partum haemorrhage. Thies‐Lagergren et al.^92^ reviewed data on more than 400,000 pregnancies in the Swedish Birth Registry. Greater risk of blood loss exceeding 1 litre in the 2 h after birth was seen in women with BMI > 25 kg m^–2^.^92^ Similarly, risk of post‐partum haemorrhage was found to be more than doubled in obese women in a study by Dalbye et al.^86^ Some of this risk is driven by a larger birthweight and head circumference in babies of obese women, leading to tearing.

## LONG‐TERM IMPLICATIONS FOR THE INFANT

Obesity during pregnancy is not restricted to carrying risk for the outcome of that pregnancy. A growing body of evidence suggests that maternal obesity is responsible for programming long‐term health and wellbeing in the growing fetus. Individuals who are exposed to maternal obesity or GDM in utero are, as adults, more likely to be obese,^93^ develop type‐2 diabetes^94^, ^95^ and die as a result of cardiovascular disease.^96^

## THE ANTENATAL PERIOD AS A TEACHABLE MOMENT

Health promotion activities that target older children and adults are generally hampered by a lack of engagement by the target population. Although certain key messages about diet and nutrition can become well‐embedded in the awareness of children and adults, compliance with such messages can be very poor. For example, the 5‐a‐day message relating to fruit and vegetable intake is almost universally known, but it fails to change behaviour across all age groups.^97^ Similarly, although most women of childbearing age in the UK are aware of the need to take folic acid supplements to prevent neural tube defects should they become pregnant, less than 40% do so according to guidelines.^98^ Although some of this is explained by around 40% of pregnancies being unplanned, there is clearly a significant proportion of women who do not follow guidelines despite being aware that they exist. The lack of engagement of some women with guidelines on lifestyle, and in particular weight, in pregnancy may be explained by a number of factors. A lack of awareness and education plays a big role, especially because most lifestyle changes need to be made before rather than during pregnancy. Having knowledge is no guarantee of action as making lifestyle changes is intrinsically difficult, especially if those changes are required without the incentive of benefitting the growing fetus. In a qualitative study of why women drink alcohol in pregnancy, Meurk et al.^99^ found that many women who did so had not appreciated the risk involved, or lived in circumstances where healthy behaviours were not the norm. The desirability of maintaining their usual social behaviours outweighed the desirability of making a lifestyle change.^99^ It is likely that the same factors apply to other unhealthy decisions made prior to and during pregnancy.

Pregnancy possibly represents the stage of life when women are most receptive to messages about health and at their most prepared to introduce lifestyle changes and has been described as a ‘teachable moment’.^100^ The motivation to change arises because women become aware that certain behaviours may put themselves and, more critically, the health of their baby at risk. Pregnancy forces a reevaluation of their role in their family and in society.^101^ Pregnancy also brings women into more contact with health professionals and literature about health and lifestyle, thereby providing routes through which the teachable moment can be capitalised upon. Unfortunately, the willingness to seek advice and information can result in women accessing sources which are not reliable. Internet sources not only have the advantage of being instantly available at all times of day and night, but also are contaminated by error and deliberate misinformation. Lynch and Nikolova^102^ found that pregnant women had a preference for finding information about their pregnancy and health on the Internet, and that they trusted what they read, although they did not question the source of the information. Internet sources are a major influence on decision making by pregnant women who are often dissatisfied by the information that they receive from health professionals, with the latter often being inaccessible to women when they have questions.^103^

The physiological response to pregnancy may in itself influence dietary behaviour right from the point of conception and this may not be conducive to making changes that control body weight gain. Nausea and vomiting are commonplace and are sometimes the first sign of conception, appearing at between 2 and 6 weeks of gestation.^104^ The nausea experienced by between 60% and 80% of women can influence food choices and the majority of women report changes in preferences for certain foodstuffs and beverages. Caffeine‐based drinks, eggs, fish, meat and fatty foods are commonly avoided, whereas intakes of carbohydrate‐rich foods tend to increase in the first trimester. Sweets, biscuits, chocolate and cakes are widely favoured, along with fruit and fruit juices.^105^, ^106^ Psychological influences are also important and some women use food to manage anxiety about their pregnancy and other negative states.^107^

Although most women undertake some degree of lifestyle change in response to becoming pregnant, if not prior to conception, the availability of a teachable moment and the health professional access that can deliver it do not guarantee that women will make the right choices. For example, although the UK Department of Health set a target of reducing the prevalence of smoking in pregnancy to 6% or less by 2022, in 2020/21, around 10% of pregnant women are still smoking by the time they give birth.^108^ Similarly, more than 40% of British women reported consuming alcohol during pregnancy, against guidelines.^109^ Generally compliance with recommendations on lifestyle change in pregnancy is greatest in women having their first baby and better educated women. Compliance is lower in younger women and those from impoverished backgrounds.^98^

In terms of managing weight and avoiding excessive weight gain in pregnancy, the opportunity to communicate clearly with women may be missed. Although, in some countries, there are clear guidelines on weight gain for pregnancy and monitoring weight is part of normal antenatal care, in the UK, the approach taken to dealing with obesity wastes an opportunity for action. Advice to women on what to eat during pregnancy, as well as what level of physical activity should be maintained, is very generalised and often poorly understood.^110^ Similarly, communication about body weight, dealing with overweight in pregnancy and what constitutes healthy weight gain is ineffectual. In the UK, the National Institute for Healthcare and Clinical Excellence (NICE) recommends that women suffering from overweight or obesity should be advised to lose weight prior to, or after, pregnancy^111^ and therefore places an emphasis on just monitoring weight gain during pregnancy. However, current clinical pathways mean that height and weight are usually only measured at the first antenatal appointment, without any further follow up. For women with a booking BMI in excess of 30 kg m^–2^, there may be a referral offered to a dietitian or other agencies so that women can receive personalised support to help manage their weight, although this is inconsistent and infrequent.^110^

Routine weighing of women at antenatal appointments has been largely abandoned in the UK, despite the importance of maintaining a healthy rate of weight gain. There are a number of reasons for this. First, NICE guidelines state that routine monitoring of women's weight without their consent and without sufficient explanation or feedback is unacceptable.^111^ There have also been studies indicating that overweight or obese women feel stigmatised, anxious or guilty when routinely weighed in pregnancy. A systematic review by Johnson et al.^112^ concluded that focusing on weight may be a barrier to optimising diet and physical activity in pregnant women. However, in a study of almost 200 women in the first trimester of pregnancy, Swift et al.^113^ found that most women would be happy to receive advice and guidance from health professionals on their weight, although only 15% of women reported having had any feedback on weight after having been weighed by their midwife, despite 31% having been overweight or obese going into pregnancy. Self‐monitoring of pregnancy weight gain was a common behaviour in this group of women, indicating that they were both interested in their weight and engaged with tracking across their pregnancy. A 2020 feasibility study in Ireland reported that women found being weighed throughout their pregnancy a positive experience and gave them reassurance with regard to the growth of their babies.^114^

Obesity is a sensitive subject, and there is evidence that, in primary care, both patients and healthcare professionals may be embarrassed and reluctant to raise the issue of body weight. Generally, it is midwives who bear the responsibility for delivering health education and promoting a healthier lifestyle in pregnancy. Although being in regular contact with women and carrying a high level of trust as a source of information, they are not well equipped for dealing with conversations about overweight.^115^, ^116^ Those conversations may be compromised by ingrained weight stigma among health professionals, which training programmes need to overcome.^117^ Midwives may lack the confidence to raise the issue of obesity and fear a hostile response from women that they are trying to form a professional bond with^112^, ^117^ and, with high workloads and time pressure, it can be difficult to maintain an awareness of unconscious bias around obese women and implement personal strategies to overcome that bias.^117^, ^118^ They also suffer from a lack of clear clinical guidelines that could enable referral to suitable personalised interventions.^118^ In the absence of regular weighing, it is also possible that the high levels of overweight in society may normalise the appearance of obesity, meaning that midwives fail to recognise women who may need intervention.^118^ Women want honest and respectful communication that provides personalised information about risk and facilitates informed lifestyle choices without scaremongering, and without proportioning blame about the causes of overweight.^119^

## STRATEGIES FOR MANAGEMENT OF WEIGHT GAIN IN PREGNANCY

Although the US Institute of Medicine recommendations on GWG^18^ are generally accepted as a good guide for achieving healthy outcomes for a pregnancy complicated by overweight or obesity, there are no clear guidelines on when or how to intervene to manage weight gain in pregnancy. In the UK, the emphasis in NICE guidelines is on achievement of a healthy weight in the interpregnancy interval^111^ and there are concerns that interventions in pregnancy could result in weight loss or inadequate weight gain, with unknown consequences for babies. Despite this, many UK NHS Trusts have implemented local services to prevent excessive weight gain, although these often work from a limited evidence base.

**Figure 4:**
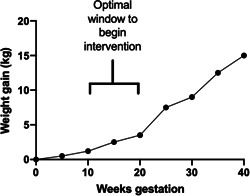
Weight gain profile for overweight women in pregnancy. The window of opportunity between antenatal booking and the rapid phase of weight gain is relatively short

As shown in Figure 4, it is likely that there is a very narrow window of time for a weight gain intervention to be initiated effectively. Across all BMI classes, weight gain in the first 20 weeks of gestation is modest (approximately 2.5 kg) and thereafter proceeds at around three to four times the early rate.^120^ The route to weight‐related complications will be established in the early‐mid gestation period because GHT can manifest from 20 weeks and GDM from 24 weeks. Because most women are not booked into antenatal services and in regular contact with health professionals until 11–12 weeks of gestation, there is a period of only a few weeks in which to introduce strategies to avoid excessive GWG before rapid gain may limit efficacy of steps taken (Figure 4).

In terms of how weight should be managed, there should be little difference in approach between pregnant and non‐pregnant individuals, except the goal for pregnancy is to allow weight gain within healthy limits rather than achieve a weight loss. Physical activity is important and previously sedentary women are advised to sit less, incorporate walking into daily life and engage with continuous exercise for up to 15 min day^–1^ (e.g. brisk walking or swimming) three times a week.^121^ Otherwise, 150 min of moderate intensity exercise per week is advised.^111^, ^121^ The unique feature of pregnancy is the availability of access to health professionals who, if appropriately trained, could advise women on weight (midwives, obstetricians, general practioners). In some cases, women may benefit from the input of specialist dietitians. Increasingly pregnant women are able to access eHealth resources that may be tailored to their weight status, such as smartphone applications.^122^, ^123^, ^124^ The latter is a particularly innovative example of taking advantage of the teachable moment in pregnancy, aiming to empower women to take control of their own health and fitness without being subjected to professional judgement and stigma. It is important that these applications provide the correct information, and also that it is tailored to the weight status of the user.

There is an extensive literature that considers the efficacy of interventions designed to limit GWG in overweight and obese women, and these interventions have produced a diverse range of sometimes conflicting outcomes. For example, Mottola et al.^125^ carried out an intervention based upon an individualised nutrition plan (2000 kcal day^–1^) coupled with a programme of walking three to four times per week. This reduced the likelihood of women exceeding recommended rates of pregnancy weight gain, although average gain was still over 12 kg as a result of excessive gain in early pregnancy. The Mighty Mums intervention was delivered in primary care, exposing women to motivational talks from midwives, food advice from dietetic consultations and prescriptions of physical activity, with women able to access services to suit their preferences.^126^ Participants had lower GWG than controls and lower post‐partum weight retention. Exposure to dietetic consultation appeared to be the main driver of success. Liu et al.^127^ implemented a very intensive programme amongst overweight and obese women in South Carolina. Participants attended an individual counselling session, 10 weeks of group sessions, had weekly phone calls from the intervention team and were given access to podcasts once a week.^127^ These contacts focused on improving dietary quality, increasing physical activity and self‐monitoring of weight. Overall, GWG was not impacted by the intervention. In African American obese women, GWG was increased in the intervention group, whereas overweight African American women had lower GWG than controls.^127^ A lighter touch intervention using the Healthy Moms smartphone app for 6 months achieved lower GWG in women who were overweight or obese, but did not bring about changes in physical activity, glycaemia or insulin resistance.^123^

Alongside the many such small‐scale interventions that focus on managing maternal weight gain in pregnancy, randomised controlled trials have sought to target either GWG or neonatal health as primary outcomes for diet or physical activity interventions in pregnancy. Two large randomised controlled trials of approaches to limiting weight gain in pregnancy and the associated risks of poor pregnancy outcomes have been extensively reported. In the LIMIT trial conducted in Australia, Dodd et al.^128^ found that a diet and lifestyle intervention reduced the risk of a birth weight above 4000 g by 18% (RR = 0.82, 95% CI = 0.68–0.99, *p* = 0.04), but the results for maternal weight gain showed no significance for the intervention group. The UPBEAT trial in the UK (2202 participants) had reduction of GDM as the primary outcome and although GWG was reduced by 0.55 kg by the intervention, GDM and other pregnancy complications were not impacted by the intervention.^129^ A meta‐analysis of 36 randomised controlled trials (RCTs) found that the success of interventions was highly dependent upon characteristics of the women recruited and whether the interventions were based upon diet, exercise or a combination of the two.^130^ Physical activity‐based interventions were generally ineffective. Interventions appear to be more effective if delivered by clinicians rather than non‐clinical staff.^131^ In women of low education, diet‐based and mixed approaches reduced the risk of excessive GWG, whereas, in highly educated women, only diet‐based interventions were successful. An earlier meta‐analysis of 44 RCTs found that interventions that mixed physical activity goals with dietary change were ineffective, but diet‐based interventions had the capacity to lower GWG and reduce risk of GHT, PE, GDM, preterm birth and labour induction.^132^ The overall analysis found that GWG reductions could greatly exceed those attained by UPBEAT.^129^ There is therefore little doubt that appropriately designed and targeted interventions can be effective tools in the management of pregnancies that are complicated by overweight and obesity. The devil lies in the detail, however, and the design, targeting and delivery of large scale, routine care to improve outcomes for overweight women is far from straightforward and is likely to be a major resource burden for local and national health services.

In 2015, we published an analysis of a pilot study for the Lincolnshire Bumps and Beyond intervention.^133^ Bumps and Beyond was available to all pregnant women in Lincolnshire whose booking BMI was 35 kg m^–2^ or greater and comprised a programme of seven sessions, which covered healthy eating, physical activity, identification of triggers that lead to unhealthy lifestyle behaviours, and relapse to old behaviours around eating and physical activity. The programme was delivered by healthy lifestyle advisors with previous experience of delivering a smoking cessation programme. The pilot study showed that the intervention reduced GWG by approximately half and resulted in a reduced prevalence of GHT and PE.^133^ Subsequent analysis (unpublished data S.C. Langley‐Evans and S. Ellis) with a bigger population confirmed that this intervention limited GWG in severely obese women and that the reduced GWG was associated with dramatically lower risk of PE (OR = 0.050 95% CI = 0.003–0.642).

There are some important lessons to be learned from the Bumps and Beyond intervention.^133^ First, it was extraordinarily successful in achieving the goal of reducing GWG by half in severely obese women and in reducing pregnancy complications, which is something that the big randomised controlled trials have failed to do. RCTs are considered to be the pinnacle of the epidemiological hierarchy, but, in the field of nutrition, where the nature of the intervention may be rather different to an RCT utilising a pharmacological agent, they do have limitations. RCTs in nutrition are often less effective than similar studies where the treatments are drugs, because the subjects may become disaffected, fail to see any clear and immediate benefit of taking part, or are disturbed by minor side‐effects and drop out, and also because the nature of the intervention may become apparent to the control group, prompting them to change their diet and behaviour in a way that detracts from the analysis. Both of these tendencies were seen with Bumps and Beyond where one‐third of participating women failed to complete the full programme and, among some women who did not take part, good control over GWG was still observed, indicating that they had chosen to make beneficial lifestyle changes without the intervention of the delivery team. Rigid RCT protocols, although most useful for researching a specific question, are likely to be less effective than a more flexible, adaptive and multimodal approach in primary care practice and this may be why Bumps and Beyond^133^ achieved results that were much greater than LIMIT^128^ or UPBEAT.^129^

Another important lesson from Bumps and Beyond was the low uptake of the programme. Only 37.5% of women invited to take part did so, and those that declined were more likely to be living in deprivation or to be experienced mothers (one or more previous pregnancies). Effective intervention strategies need to find a way of including hard to reach social groups because these are the women at greatest risk. The final lesson to be learned from Bumps and Beyond comes from the experience of rolling out the same programme in a different setting. When the programme was operated in the neighbouring county of Nottinghamshire, there was no effect on either GWG or pregnancy outcomes, in contrast to the success in Lincolnshire. This may be explained by greater ethnic diversity in the population that was targeted, a more open recruitment strategy (women with BMI > 30 kg m^–2^), or the new delivery team having a different skillset and approach to delivering the sessions. It is likely that successful intervention will require pregnant women to be given a more bespoke and culturally sensitive experience founded on a close partnership with a health professional trained in behaviour change techniques. There is evidence that training midwives in healthy conversation skills and extending appointment times enables the use of those skills and allows them to be effective in raising issues around diet and physical activity with pregnant women.^134^ Downs et al.^135^ explored the feasibility of such an approach in a pilot study. Women in their trial were given 60 min with a dietitian per week between 8 and 20 weeks of gestation and this was either maintained in those who kept GWG within set limits, or was increased in those who exceeded limits. In effect, this was an intervention delivered at adaptive doses to suit the needs of each woman involved. Compliance was 87%, indicating that this intensive input was largely acceptable to the women. Although the intervention reduced GWG by 21%, the trial was too small to determine a statistically significant effect.^135^

## CONCLUSIONS

As the global prevalence of overweight and obesity continues to increase year on year, the associated threat to the health and wellbeing of pregnant women and their infants, as well as the cost of managing adverse pregnancy outcomes, is becoming increasingly significant. It is clear that there are approaches that can be taken to reduce the risk of poor outcomes, although, for these to be successful in primary care, investment will be needed for both the training of health professionals and the delivery of interventions suited to the needs of individual women. For the greatest effect, conversations about weight management need to occur in the first trimester, which, although challenging, is likely to be the best time to capitalise on the teachable moment that early pregnancy offers. For greatest impact, the future needs of antenatal weight management in primary care may be best delivered through eHealth approaches.

## CONFLICT OF INTERESTS

The authors declare that there are no conflicts of interest.

## AUTHOR CONTRIBUTIONS

All authors contributed equally to the writing of this review.
